# Two-Step Joint Optimization with Auxiliary Loss Function for Noise-Robust Speech Recognition

**DOI:** 10.3390/s22145381

**Published:** 2022-07-19

**Authors:** Geon Woo Lee, Hong Kook Kim

**Affiliations:** 1 AI Graduate School, Gwangju Institute of Science and Technology, Gwangju 61005, Korea; geonwoo0801@gist.ac.kr; 2 School of Electrical Engineering and Computer Science, Gwangju Institute of Science and Technology, Gwangju 61005, Korea

**Keywords:** joint optimization, auxiliary loss function, speech enhancement, noise-robust speech recognition

## Abstract

In this paper, a new two-step joint optimization approach based on the asynchronous subregion optimization method is proposed for training a pipeline model composed of two different models. The first-step processing of the proposed joint optimization approach trains the front-end model only, and the second-step processing trains all the parameters of the combined model together. In the asynchronous subregion optimization method, the first-step processing only supports the goal of the front-end model. However, the first-step processing of the proposed approach works with a new loss function to make the front-end model support the goal of the back-end model. The proposed optimization approach was applied, here, to a pipeline composed of a deep complex convolutional recurrent network (DCCRN)-based speech enhancement model and a conformer-transducer-based ASR model as a front-end and a back-end, respectively. Then, the performance of the proposed two-step joint optimization approach was evaluated on the LibriSpeech automatic speech recognition (ASR) corpus in noisy environments by measuring the character error rate (CER) and word error rate (WER). In addition, an ablation study was carried out to examine the effectiveness of the proposed optimization approach on each of the processing blocks in the conformer-transducer ASR model. Consequently, it was shown from the ablation study that the conformer-transducer-based ASR model with the joint network trained only by the proposed optimization approach achieved the lowest average CER and WER. Moreover, the proposed optimization approach reduced the average CER and WER on the Test-Noisy dataset under matched noise conditions by 0.30% and 0.48%, respectively, compared to the approach of separate optimization of speech enhancement and ASR. Compared to the conventional two-step joint optimization approach, the proposed optimization approach provided average CER and WER reductions of 0.22% and 0.31%, respectively. Moreover, it was revealed that the proposed optimization approach achieved a lower average CER and WER, by 0.32% and 0.43%, respectively, than the conventional optimization approach under mismatched noise conditions.

## 1. Introduction

In recent years, the development of deep learning technology has substantially improved the performance of speech processing algorithms such as automatic speech recognition (ASR) [1,2], speech separation [3], and speech enhancement [4]. Among them, ASR has been popularly deployed for voice-enabled information retrieval using artificial intelligence (AI) speakers and chatbots [5,6,7,8]. It has also been used for the transcription of social media videos [9] and video conferencing [10,11]. Traditionally, an ASR system is composed of three modules: a feature extractor for representation of the speech signal, an acoustic model for mapping acoustic features to linguistic units, and a language model regarding the grammar, lexicon, etc. [12,13]. Recent state-of-the-art ASR systems are generally based on an end-to-end neural network structure that uses a sequence-to-sequence training strategy to transduce a speech signal to the corresponding word sequence [14,15,16,17,18,19,20,21,22,23]. An end-to-end ASR system is typically constructed as a form of encoder–decoder structure, where the encoder and decoder correspond to an acoustic model and a language model, respectively [14,15,16,17,18,19,20,21].

Some end-to-end ASR systems have the same neural network architecture for both the encoder and the decoder. The initial versions of the end-to-end ASR system were realized using recurrent neural network (RNN) modules for both the encoder and decoder [14]. They achieved better performance than neural network-based modular-structured ASR systems [15]. However, the information flow in such an encoder–decoder structure is restricted from the last layer of the encoder to the first layer of the decoder. This limits the representation of the contextual information for the alignment between speech signals and text [16]. Such a long-term dependency problem can be mitigated by applying an attention mechanism to construct a context vector between the encoder and decoder. The RNN-based encoder–decoder architecture with attention was shown to be better than that without any attention, but the long-term dependency problem still occurred in the RNN layers themselves [16,17]. Thus, the transformer-based architecture was proposed to address the long-term dependency problem. As expected, the transformer-based ASR systems provided better performance than the RNN-based ones, but they had another limitation in that the local contextual information of speech signals was not captured by the transform [18,19]. Therefore, the state-of-the-art end-to-end ASR systems employed a conformer [20] or the ContextNet [21] instead of a conventional transformer. 

Other end-to-end ASR systems have been realized by constructing the decoder with a different architecture from the encoder. In other words, an attention-based architecture described above is used only for the encoder of the end-to-end ASR system. Instead, the decoder of the end-to-end ASR system is constructed by connectionist temporal classification (CTC) [14] or a neural transducer model [20,24]. In [24], the RNN-transducer (RNN-T) model was proposed by using an RNN and a neural transducer as the encoder and decoder, respectively. The RNN-T model showed superior ASR performance to the end-to-end ASR systems using CTC and an attention-based decoder. This was because the RNN-T model could overcome a well-known issue associated with the conditional independence assumption of CTC [14]. The performance of the RNN-T model was further improved by replacing the RNN structure with a conformer, which was called the conformer-transducer [24]. This was because the conformer was better than the RNN in capturing both global and local contextual information as in the encoder–decoder structure. 

Nevertheless, the end-to-end ASR systems described above degraded the performance when speech signals were recorded via a close-talking microphone or under low signal-to-noise ratio (SNR) conditions [22,23]. This is because the presence of background noise in the real world could severely distort speech signals [25,26,27]. To mitigate this issue, many research works have attempted to incorporate a speech enhancement technique into ASR. 

Traditionally, speech enhancement techniques have been developed to enhance speech quality for voice communications equipped with a single-channel microphone or a multi-channel microphone array [4,26,27,28,29,30,31,32,33]. To overcome the noise robustness of ASR, the developed speech enhancement algorithm can be used as a front-end of ASR. Among various types of speech enhancement algorithms, deep learning-based speech enhancement models achieved a superior performance compared to conventional statistical methods [4]. In particular, U-Net-based speech enhancement models showed better performance than other neural network architectures [28]. Since then, the U-Net architecture has been evolving by having recurrent structures and/or attention mechanisms to represent local and global time-dependent information of speech signals. A convolutional recurrent network (CRN)-based speech enhancement model was proposed to improve the conventional U-Net-based one, where long short-term memory (LSTM) layers were used as a bottleneck layer of the U-Net [29]. Furthermore, the CRN was extended into a deep complex convolutional recurrent network (DCCRN) to improve speech enhancement performance under low signal-to-noise ratio (SNR) conditions, where the DCCRN dealt with a complex spectrum to differentiate the phase of speech from that of the noise signal [30]. 

However, these speech enhancement models could generate enhanced speech signals with unintentional artifacts. These artifacts act as mismatched conditions for ASR, which causes ASR performance degradation. This happens because the speech enhancement model is trained without regard to ASR. To address the artifact issue when speech enhancement is used as a front-end of ASR, a multi-condition training approach can be applied to train ASR. Thus, the training dataset for ASR must include all the speech signals before and after speech enhancement to accommodate the artifacts contaminating the enhanced speech signals. Multi-condition training was reported to have improved ASR performance, compared to training ASR only using speech signals prior to speech enhancement [27,31], but the performance improvement was limited. This was because the enhanced signals were generated from the speech enhancement model that was trained without accounting for the characteristics of the ASR model following it [34].

The use of a joint optimization framework between a speech enhancement and an ASR model has also been investigated [34,35,36,37]. Specifically, a speech enhancement model and an ASR model can be used as a front-end and a back-end module, respectively, to construct a pipeline for joint optimization. Then, the entire model parameters in this pipeline can be optimized together by considering the speech quality loss and the ASR loss. For example, a joint optimization model composed of a generative adversarial network-based speech enhancement and a transformer-based ASR was proposed to improve the ASR performance, where the loss function was devised by combining an ASR loss and an enhancement loss [34]. Additionally, a pipeline for joint optimization composed of a bi-directional long-short term memory (BiLSTM)-based speech enhancement and a conformer-based ASR was proposed [35,36]. In [37], joint optimization was performed by constructing a pipeline using the model parameters of the front-end and the back-end model, where the front-end and back-end models were already trained individually with their own loss functions. 

However, such joint optimization suffers from the fact that each task in the pipeline has its own goal such as enhancing speech or improving ASR performance. Consequently, individual tasks might be competing against the maximization of each other’s goals. This could lead to difficulty in convergence owing to different gradient directions [38]. In [38], an asynchronous subregion optimization method between speech enhancement and speaker recognition as a front-end and a back-end model, respectively, was proposed to resolve the aforementioned issue. The model parameters of the speech enhancement model alone were first updated using a speech enhancement dataset with the loss function for speech enhancement. Next, a pipeline for joint optimization was constructed by concatenating the updated speech enhancement model and the speaker recognition model, and then all the model parameters were jointly updated using a combined loss function for speech enhancement and speaker recognition. This refined joint optimization procedure works better than jointly optimizing the two models together right from the beginning, but it has a drawback that the model parameters of the front-end model might be updated only towards the promotion of the speech enhancement goal and not the ASR performance, which could limit the performance improvement of the back-end model.

Therefore, we present a new joint optimization approach based on the asynchronous subregion optimization method in [38]. Specifically, while a speech quality objective function is only utilized to update the front-end model or the speech enhancement model [38], we propose a loss function for speech enhancement so that it can also take into account ASR performance for updating the speech enhancement alone. The proposed loss function for speech enhancement is composed of the speech quality objective function and an auxiliary ASR loss. To this end, we first extracted the enhanced speech by applying the speech enhancement model to noisy speech. Given that the goal of the speech enhancement model is to estimate the clean version of the noisy speech, the SNR between the clean and the enhanced speech becomes the speech quality objective function. To obtain the auxiliary ASR loss, the clean and the enhanced speech are separately passed to the encoder block in the end-to-end ASR model, resulting in the encoder output for each input speech. Next, the Euclidean distance between the two encoder outputs was computed, which is defined as the auxiliary ASR loss. As the auxiliary loss should be small if the ASR model has worked well, this loss can be interpreted as the ASR performance, and therefore the speech enhancement model is expected to be directed to support the ASR goal.

The main contribution of this work is three parts as follows: (1) An auxiliary ASR loss function was proposed to resolve the difficulty in convergence owing to different gradient directions between the speech enhancement and ASR models; (2) It demonstrated that the auxiliary ASR loss function can improve the ASR performance of the pipeline constructed by the speech enhancement and ASR models when it is employed in the proposed two-step joint optimization approach. In addition, (3) an ablation study was carried out to examine the effectiveness of the proposed joint optimization approach on each of three different modules in the conformer-transducer-based ASR model.

The rest of this paper is organized as follows: Section 2 briefly reviews the speech enhancement and ASR models employed in this work. Then, Section 3 proposes a two-step joint optimization approach, focusing on the loss function for the speech enhancement model for ASR. Section 4 evaluates the ASR performance of the joint optimization employing the proposed loss function and compares it to other joint optimization approaches under matched and mismatched noise conditions. Finally, we conclude the paper in Section 5.

## 2. Review of Speech Enhancement and ASR Models

This section reviews a speech enhancement model and an ASR model employed as the front-end and back-end models, respectively, for joint optimization to improve ASR performance.

### 2.1. Deep Complex Convolutional Recurrent Network-Based Speech Enhancement Model

**Figure 1:**
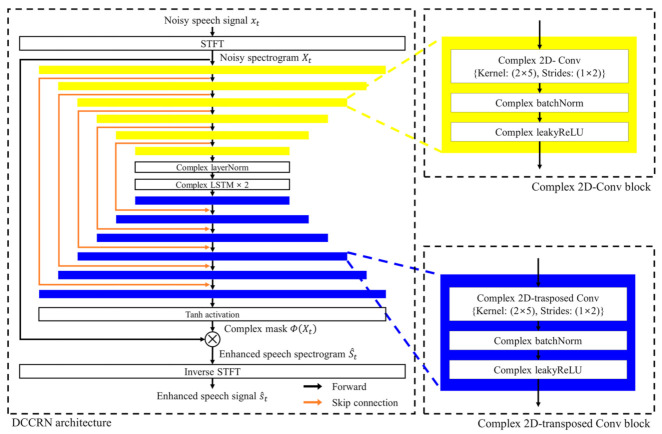
Architecture of the deep complex convolution recurrent network (DCCRN)-based speech enhancement, where ⊕ is the component-wise vector multiplication operator [30].

Figure 1 shows the architecture of the DCCRN-based speech enhancement model [30], which exhibits a state-of-the art performance in terms of objective and subjective speech quality metrics. As shown in the figure, the DCCRN architecture is composed of a causal complex encoder and decoder with two LSTM layers between the encoder and the decoder blocks. The complex encoder consists of six complex convolutional blocks, and each block is sequentially composed of a complex 2D convolutional layer, batch normalization, and rectified linear units (ReLUs), displayed as yellow boxes in the figure. Consequently, the output of the last complex 2D convolutional block in the complex encoder represents the feature of the noisy speech signal. In parallel, the complex decoder, which is represented by the blue boxes in this figure, is also composed of six complex 2D transpose convolution blocks, which operate in a reverse sequence to those of the complex encoder block. The output of each transpose convolution block is then concatenated with that of its corresponding convolution block, and the concatenated output is brought as the input feature for the next transpose convolution block. Furthermore, a hyperbolic tangent (tanh) activation function is applied to the output of the last convolution block in the complex decoder. 

Given a clean speech signal, ***s***, it is segmented into consecutive frames with an overlap size of 160 samples, where the frame size is 512 at a sampling rate of 16 kHz. Then, the noisy speech signal, $x_{t}$, at the *t*-th frame can be expressed as $x_{t}=s_{t}+d_{t}$, where $d_{t}$ is a noise signal added at the *t*-th frame. By applying a 512-point short-time Fourier transform (STFT) to $x_{t}$, a noisy spectrum, $X_{t}$, is obtained. Next, the output of the DCCRN-based speech enhancement model, $\Phi \left(X_{t}\right),\;$ is used to mask the noisy input complex spectrogram, $X_{t},$ resulting in the estimate of the clean speech spectrum at the *t*-th frame, ${\hat{S}}_{t}.$ To estimate a time-domain clean speech at the *t*-th frame, ${\hat{s}}_{t},\;$ a 512-point inverse STFT (ISTFT) followed by the overlap–add method is applied to ${\hat{S}}_{t}.$ Finally, the estimated clean speech, ${\hat{s}}_{t},$ is used as an input feature of the following ASR model.

### 2.2. End-to-End Conformer-Transducer-Based Speech Recognition Model

**Figure 2:**
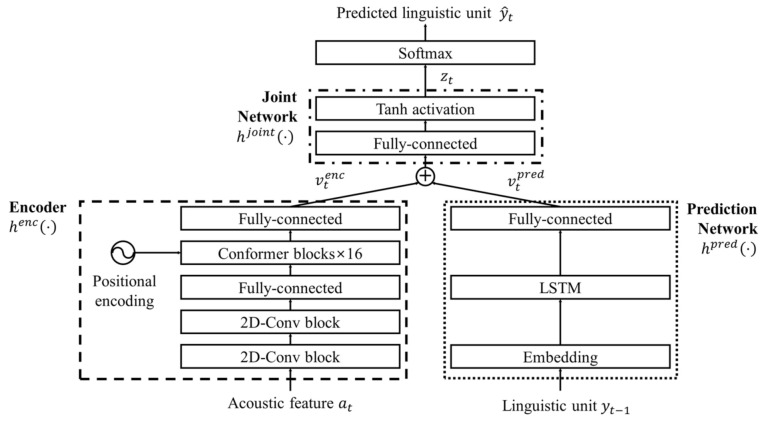
Architecture of the conformer-transducer-based speech recognition model, where ⊕ is the vector-addition operator [20].

Figure 2 shows the architecture of a conformer-transducer ASR model that is identical to the model in [20]. For a given pair of speech signal and sentence for training ASR, the speech signal, $s=\left\{s_{1},\;s_{2},\cdots ,s_{T}\right\}$, can be transformed into an acoustic feature vector sequence, $a=\left\{a_{1},\;a_{2},\cdots ,a_{T}\right\}$, where *T* is the total number of frames in s. Note here that the 80-dimensional log-mel spectrum analysis is applied to $s_{t}$ to obtain $a_{t}$. Similarly, a linguistic unit sequence with a length of U can be expressed as $y=\left\{y_{1},\;y_{2},\cdots ,y_{U}\right\}$, where $y_{i}$ is the *i*-th linguistic unit in the target text sequence. 

As shown in the figure, the conformer-transducer-based ASR model consists of an encoder, a prediction network, and a joint network. The encoder, $h^{enc}\left(\cdot \right),$ tries to represent an acoustic feature, $v_{t}^{enc},$ from a feature vector, $a_{t},\;$ as

(1) $$v_{t}^{enc}=h^{enc}\left(a_{t}\right).$$

where $h^{enc}\left(\cdot \right)$ is composed of two 2D convolutional blocks, one fully connected layer, sixteen conformer blocks, and one fully connected layer, sequentially. In parallel, the prediction network, $h^{pred}\left(\cdot \right),$ represents a linguistic feature, $v_{t}^{pred}$, from the target or a predicted linguistic unit at time $\left(t-1\right)$, $y_{t-1},$ for training or inference, respectively. In other words, $h^{pred}\left(\cdot \right)$ consists of an embedding layer, an LSTM layer, and a fully connected layer. The output of the prediction network is represented as

(2) $$v_{t}^{pred}=h^{pred}\left(y_{t-1}\right)$$

where $v_{1}^{pred}$ is predicted from the embedding vector corresponding to the begin-of-sentence token, $y_{0}.$ In fact, $v_{t}^{pred}$ contains all the linguistic information from the beginning of the sentence up to the linguistic unit at time $\left(t-1\right)$, because an LSTM network is used in the prediction network. 

After that, the joint network, $h^{joint}\left(\cdot ,\cdot \right)$, adds the encoder output, $v_{t}^{enc}$, in (1) and the prediction network output, $v_{t}^{pred}$, in (2) to produce logits, $z_{t}$, as

(3) $$z_{t}=h^{joint}\left(v_{t}^{enc},v_{t}^{pred}\right)$$

where $h^{joint}\left(\cdot ,\cdot \right)$ is realized by a fully connected layer and a tanh activation function. To identify the predicted linguistic unit,$\;{\hat{y}}_{t}$, the output node index, ${\hat{i}}_{t}$, in which $z_{t}$ is the highest value, is searched. Then, ${\hat{i}}_{t}$ is modified as ‘blank index’ if ${\hat{i}}_{t}={\hat{i}}_{t-1}$. Next, ${\hat{y}}_{t}$ is assigned as ‘blank’ if ${\hat{i}}_{t}$ is ‘blank index’; otherwise, ${\hat{y}}_{t}$ is the linguistic unit representing the ${\hat{i}}_{t}$-th output node. In addition, for training, the number of linguistic units included in $\left\{{\hat{y}}_{1},\;{\hat{y}}_{2},\cdots ,{\hat{y}}_{t}\right\}$ is counted, which is denoted as $c_{t}$. Next, $y_{c_{t}}$ is sent to the input of the prediction network for further training. 

Note here that the hyper-parameters of the conformer-transducer-based ASR model are identically set to those in [20]. That is, the numbers of input and output nodes for the encoder are 80 and 144, respectively, and the numbers of input and output nodes for the prediction network are 512 and 320, respectively. In addition, the number of input nodes for the joint network is 1024, but the number of output nodes is 1000 because the total number of linguistic units for ASR is 1000. Finally, the conformer-transducer-based ASR model described thus far is utilized as the back-end module in the pipeline, and the text transcription is predicted using the enhanced speech from the front-end module.

## 3. Proposed Joint Optimization Approach for Noise-Robust ASR

**Figure 3:**
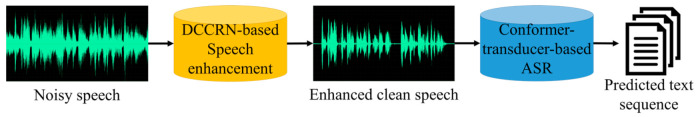
Block diagram of the noise-robust ASR used in this experiment.

This section proposes a two-step joint optimization approach for ASR. Figure 3 shows the block diagram of the pipeline employed in this paper. As shown in the figure, the enhanced speech is generated from the noisy speech by applying the DCCRN-based speech enhancement model, as described in Section 2.1. Then, this enhanced speech is used as the input feature of the conformer-transducer-based ASR model to predict a text sequence, as described in Section 2.2. 

To apply the proposed two-step joint optimization approach to the pipeline, it is assumed that the DCCRN-based speech enhancement and conformer-transducer-based ASR models are already individually trained from scratch. Then, the proposed two-step joint optimization is performed. In the first step, a speech quality loss between the clean and the enhanced speech is computed. In parallel, an auxiliary ASR loss is also computed after processing the clean speech and the enhanced speech separately by the encoder block of the conformer-transducer ASR model. Then, the two losses are combined and used for optimizing the DCCRN-based speech enhancement model. In the second step, all the model parameters in the pipeline are optimized together by using the speech quality loss and the ASR loss that is different from the auxiliary ASR loss used in the first step. The following subsections describe the detailed procedures of the first and second steps of the proposed joint optimization. 

### 3.1. First-Step Processing in the Proposed Joint Optimization

**Figure 4:**
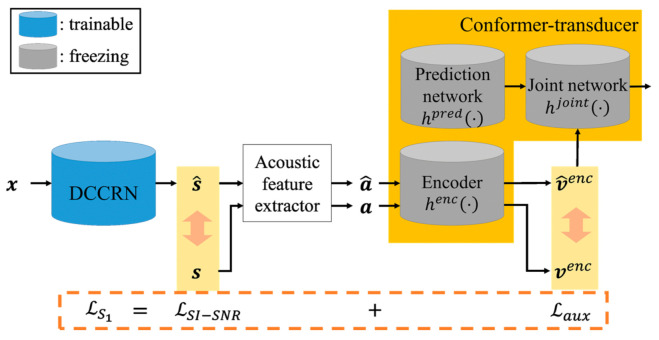
Block diagram of computing the first-step loss of the proposed two-step joint optimization approach.

Figure 4 shows the block diagram of computing a combined loss used for the first-step processing of the proposed two-step joint optimization approach. For the given clean and noisy speech at the *t*-th frame, $s_{t}$ and $x_{t}$, the enhanced speech, ${\hat{s}}_{t},$ is first extracted by applying the DCCRN-based speech enhancement model to $x_{t}$. Additionally, the clean and enhanced acoustic features at the *t*-th frame, $a_{t}\;$ and ${\hat{a}}_{t}$, are generated from $s_{t}$ and ${\hat{s}}_{t}$ by using an acoustic feature extractor, respectively. In this paper, the 80-dimensional log-mel spectrum analysis is used as the acoustic feature extractor. 

Then, the scale-invariant SNR (SI-SNR) between the clean and the enhanced speech, $L_{SI-SNR}$, is computed as a speech quality loss, which is defined as [30]

(4) $$L_{SI-SNR}=-10\overset{T}{\underset{t=1}{\sum }}\log_{10}\left(\frac{∥s_{t}^{target}{∥}^{2}}{∥{\hat{s}}_{t}-s_{t}^{target}{∥}^{2}}\right)$$

where $s_{t}^{target}=\langle {\hat{s}}_{t},\;s_{t}\rangle \cdot s_{t}/∥s_{t}{∥}^{2}$ is a scaled version of $s_{t}$, and *T* denotes the total number of frames for each training batch. Note here that the conventional asynchronous subregion optimization method only used $L_{SI-SNR}$ for the optimization of the speech enhancement [38]. In this case, the model parameters of the speech enhancement model could be updated in a direction towards improving speech enhancement performance but not ASR performance. Therefore, we considered another type of loss function to enforce the speech enhancement model to be trained for improving ASR performance.

To this end, an auxiliary ASR loss is computed based on the assumption that the output of the encoder in the conformer-transducer-based ASR model is regarded as a high-level acoustic feature for speech recognition [21]. Specifically, each of the clean and enhanced acoustic features, $a_{t}\;$ and ${\hat{a}}_{t}$, is passed to the encoder block in the ASR model, resulting in two encoder outputs such as $v_{t}^{enc}=h^{enc}\left(a_{t}\right)$ and ${\hat{v}}_{t}{}^{enc}=h^{enc}\left({\hat{a}}_{t}\right)$ by using Equation (1) in Section 2.2. Then, the auxiliary ASR loss is computed as the Euclidian distance between these encoder outputs, $v_{t}^{enc}$ and ${\hat{v}}_{t}{}^{enc}$, such as

(5) $$L_{aux}=\overset{T}{\underset{t=1}{\sum }}\sqrt{{\left(v_{t}^{enc}-{\hat{v}}_{t}^{enc}\right)}^{2}}.\;$$

Finally, the DCCRN-based speech enhancement model is trained using the combined loss, $L_{s_{1}},$ that is defined as

(6) $$L_{s_{1}}=\alpha_{1}\cdot L_{SI-SNR}+\left(1-\alpha_{1}\right)\cdot L_{aux}$$

where $\alpha_{1}$ is a control parameter for placing emphasis on the speech enhancement quality or ASR performance. Based on exhaustive experiments using various values of $\alpha_{1}$, it was set to 0.5 in this study.

### 3.2. Second-Step Processing in the Proposed Joint Optimization

For the second-step processing of the proposed two-step joint optimization approach, the model parameters of the DCCRN-based speech enhancement model are replaced with those obtained from the first-step processing. Next, the speech enhancement and ASR models are optimized together with a second-step loss that considers the speech quality as well as ASR performance.

**Figure 5:**
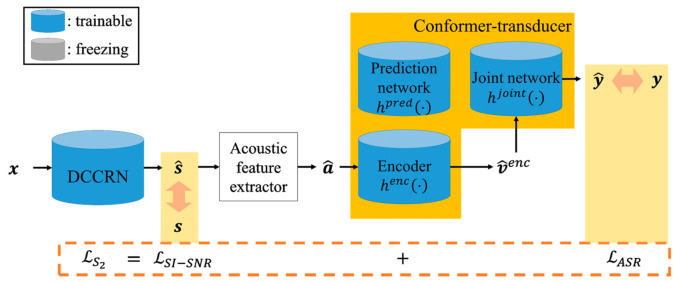
Block diagram of computing the second-step loss of the proposed two-step joint optimization approach.

Figure 5 shows the procedure of computing the second-step loss of the proposed two-step joint optimization approach. To train the conformer-transducer-based ASR model, a log posterior of the previously predicted linguistic unit, ${\hat{y}}_{t}$, is first computed from the softmax output of ${\hat{z}}_{t}$ at the node index corresponding to $y_{c_{t}}$, as described in Equation (3). This probability can be denoted as $P\left({\hat{y}}_{t}|a_{t},y\right)$ because ${\hat{y}}_{t}$ is predicted when the acoustic feature, $a_{t}$, and target linguistic sequence, y, are given [24]. Thus, the ASR loss, $L_{ASR},$ is computed by summing the negative log posteriors for all the speech frames, which is defined as

(7) $$L_{ASR}=-\overset{T}{\underset{t=1}{\sum }}\log P\left({\hat{y}}_{t}|a_{t},y\right).$$

Since the second step optimizes the ASR model with a support of speech enhancement, the ASR loss in Equation (7) is combined with the speech quality loss, $L_{SI-SNR}$, in Equation (4). Thus, the combined loss used in the second step, $L_{s_{2}},$ is defined as

(8) $$L_{s_{2}}=\alpha_{2}\cdot L_{SI-SNR}+\left(1-\alpha_{2}\right)\cdot L_{ASR}.$$

where $\alpha_{2}$ is also a parameter for trading off the weight loss ratio between the speech quality and ASR performance, and it was set to 0.05 based on the experiments.

The procedure of the proposed two-step joint optimization approach is summarized in Algorithm 1. For the given sets of hyper-parameters and pretrained models, the algorithm was applied on a basis of mini-batches. Specifically, lines 5 and 6 of the algorithm denote the first-step and second-step processing of the proposed optimization approach, respectively.
**Algorithm 1:** Two-Step Joint Optimization Procedure with Auxiliary ASR Loss.**Require**: m, batch size. n, the number of iterations. $\alpha_{1}$, auxiliary ASR loss weight. $\alpha_{2}$, loss weight ratio between speech enhancement and ASR. $\theta_{SE}$, speech enhancement learnable parameters. $\theta_{ASR}$, ASR learnable parameters.1:**for**1,⋯,n**do**2:   Mini-batch of m clean speech frames $\left\{s^{\left(1\right)},\cdots ,s^{\left(m\right)}\right\}$ and corresponding acoustic features $\left\{a^{\left(1\right)},\cdots ,a^{\left(m\right)}\right\}$
3:   Mini-batch of m enhanced speech frames $\left\{{\hat{s}}^{\left(1\right)},\cdots ,{\hat{s}}^{\left(m\right)}\right\}$ and corresponding acoustic features $\left\{{\hat{a}}^{\left(1\right)},\cdots ,{\hat{a}}^{\left(m\right)}\right\}$
4:   Mini-batch of m linguistic units $\left\{y^{\left(1\right)},\cdots ,y^{\left(m\right)}\right\}$
5:   First-Step Processing: Update the speech enhancement parameters using SI-SNR and auxiliary ASR loss
$\nabla_{\theta_{SE}}\frac{1}{m}\overset{m}{\underset{i=1}{\sum }}\left(\alpha_{1}\cdot {ℒ}_{SI-SNR}\left(s^{\left(i\right)},{\hat{s}}^{\left(i\right)}\right)+\left(1-\alpha_{1}\right)\cdot {ℒ}_{aux}\left(a^{\left(i\right)},{\hat{a}}^{\left(i\right)}\right)\right)$6:   Second-Step Processing: Update both speech enhancement and ASR parameters using SI-SNR and ASR loss
$\nabla_{\theta_{SE},\;\theta_{ASR}}\frac{1}{m}\overset{m}{\underset{i=1}{\sum }}\left(\alpha_{2}\cdot {ℒ}_{SI-SNR}\left(s^{\left(i\right)},{\hat{s}}^{\left(i\right)}\right)+\left(1-\alpha_{2}\right)\cdot {ℒ}_{ASR}\left(a^{\left(i\right)},y^{\left(i\right)}\right)\right)$7:**end for**


**Figure 6:**
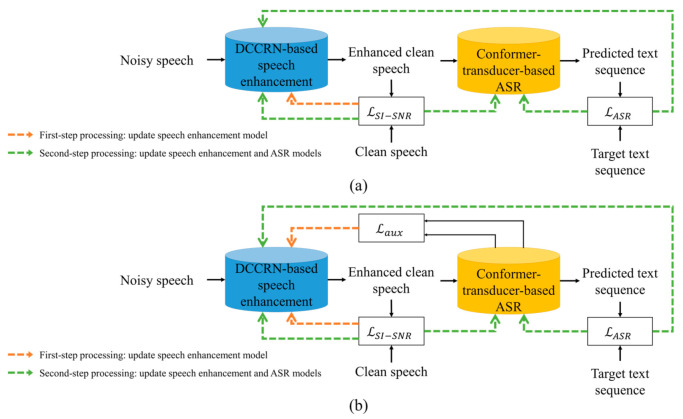
Block diagram comparison of the joint optimization approaches between (**a**) the conventional joint optimization [38] and (**b**) the proposed two-step joint optimization, applied to the noise-robust ASR pipeline composed of DCCRN-based speech enhancement and conformer-transducer-based ASR models.

Next, Figure 6 compares the procedure of the proposed two-step joint optimization approach with that of the conventional approach proposed in [38]. As shown in Figure 6a, the first-step processing in the traditional joint optimization approach is applied to optimize the speech enhancement model with the speech quality loss only, while the second-step processing is applied to the speech enhancement and ASR models with the speech quality loss and ASR loss, respectively. Therefore, only using the quality loss for training the speech enhancement model training causes the model training convergence to slow in the conventional joint optimization approach. On the other hand, the proposed joint optimization approach shown in Figure 6b can mitigate the difficulty in convergence by combining the auxiliary ASR loss to the quality loss for training the speech enhancement model.

## 4. Experiments

This section evaluated the performance of the conformer-transducer ASR model applied using the proposed two-step joint optimization approach and compared it with the performance using multi-condition training [27] and the conventional joint optimization approaches [37,38]. In addition, an ablation study was performed to examine the effectiveness of the proposed joint optimization approach according to each processing block of the conformer-transducer ASR model.

### 4.1. Experimental Setup

In this experiment, we prepared 281,241 clean speech utterances spoken by 2338 speakers excerpted from train-clean-100, train-clean-360, and train-other-500 in the LibriSpeech database [39] as the clean training dataset. Here, the total length of all the utterances was 960 h, and the average length per utterance was measured as 12.3 s. In order to simulate various noise conditions, we constructed a noisy training dataset by using the wideband noise dataset released by the third Deep Noise Suppression (DNS) challenge [40]. Actually, the noise dataset was collected from Audioset, Freesound, and the Diverse Environments Multi-Channel Acoustic Noise Database (DEMAND), including around 150 different noise types that could exist during voice over Internet protocol (VoIP) services. The noisy speech utterances were obtained by mixing each clean speech utterance with the noise data that were randomly selected from the noise dataset. To simulate different noise levels, the mixing ratio between clean speech and noise was controlled so that the SNR ranged from −5 to 20 dB at a step of 1 dB. 

To validate the model training, we used the Dev-Clean dataset in the LibriSpeech database. The Dev-Clean dataset was composed of 2703 clean speech utterances spoken by 40 speakers, whose amount was 5.4 h in total and whose average length was measured as 7.4 s. Similar to the training dataset described above, the noisy version of the Dev-Clean was obtained by adding the wideband DNS noise to each of the clean utterances in Dev-Clean, which is referred to as the Dev-Noisy dataset in this paper.

Next, to evaluate the ASR performance of the pipeline optimized by the proposed two-step joint optimization approach, the Test-Clean dataset in the LibriSpeech database was used, where there were 2620 clean speech utterances spoken by 40 speakers, with an average length of 7.4 s. To create the noisy version of Test-Clean, which is referred to as the Test-Noisy dataset, we randomly added the wideband DNS noise to each of the clean utterances in Test-Clean under SNR conditions in the range of −5–20 dB at a 1 dB step.

Moreover, we constructed another test dataset to evaluate the performance of the ASR models under mismatched noise conditions. To this end, we prepared a noise dataset from the NOISEX92 database. Specifically, we collected noise signals belonging to 14 different noise types, such as white, pink, babble, factory1, factory2, buccaneer1, buccaneer2, f16, destroyerengine, destroyerops, leopard, m109, volvo, and hfchannel. Note here that these noise types were not overlapped with the noise types in the wideband DNS noise dataset. Similar to Test-Noisy, we randomly added 1 of the 14 different noise signals to each of the utterances in Test-Clean under SNR conditions in the range of −5–20 dB at a 1 dB step, which is referred to as the Test-Noisy-Mismatched dataset. Note here again that the Test-Noisy and Test-Noisy-Mismatched datasets had different types of noise, while the number of utterances in the Test-Noisy dataset was the same as that of the Test-Noisy-Mismatched dataset.

For a fair comparison, the architecture and hyper-parameters of the DCCRN-based speech enhancement model were identically set to those in [30]. In other words, the input feature was a complex spectrum obtained by applying a 512-point STFT to each noisy speech frame with a frame size of 25 ms and a frame shift size of 10 ms. The number of convolutional blocks for the encoder and decoder was set to six each, and these six convolutional blocks had varying numbers of channels such as (32, 64, 128, 128, 256, 256) with a kernel size of 5 × 2 and stride size of 2 × 1.

Similarly, the architecture and hyper-parameters of the conformer-transducer-based ASR model were also identically set to those in the small-sized conformer as described in [20]. As an input feature for the ASR model, an 80-dimensional log-mel spectrum was extracted for the conformer-transducer ASR. In addition, the number of input feature vectors was increased by using the SpecAugment technique [41]. As a target feature, the linguistic units for transcribing target texts consisted of a special token and 1000 linguistic units generated by the unigram language model algorithm [42]. 

All the models including individual training and joint optimization were trained using the Adam optimizer. To adjust the learning rate, the plateau learning rate scheduler [43] and the warmup learning rate scheduler techniques with 40,000 warmup steps [44] were applied to pre-train the DCCRN-based speech enhancement model and the conformer-transducer-based ASR model, respectively [45]. For the proposed joint optimization approach, the exponential moving average (EMA) technique with a decay of 0.9999 was applied to update all the model parameters, because the prediction ability of the speech enhancement model changed from iteration to iteration so that enhanced speech signals from the speech enhancement model were different before and after each iteration [46]. This resulted in a fluctuation in loss according to the learning iterations. This issue was mitigated by smoothing the gradients using EMA. All the training and optimization approaches were implemented in Python 3.8.10 using TensorFlow 2.7.0 and Horovod 0.23.0 [47,48], and all the experiments were conducted on an Intel(R) Xeon(R) Gold 6226R workstation using four Nvidia RTX 3090s.

### 4.2. Performance Evaluation under Matched Noise Conditions

The performance of each ASR model obtained by various optimization approaches was evaluated by measuring the character error rate (CER) and word error rate (WER). The ASR models compared here were (1) ASR-only trained using the clean training dataset; (2) ASR-only trained using the noisy training dataset; (3) a combination of the speech enhancement (SE) and ASR models (denoted as SE-ASR) after each of the two models was separately trained using the noisy training dataset; (4) a combined model of the SE and ASR models (denoted as SE+ASR) trained by a conventional joint optimization as in [37]; (5) SE+ASR trained using a conventional two-step joint optimization as in [38]; and (6) SE+ASR trained using the proposed two-step joint optimization. Note that all the combined models from (3) to (6) were trained using the noisy training dataset.

Table 1 compares the average CER and WER of the ASR models trained using various joint optimization approaches evaluated on four different datasets such as Dev-Clean, Dev-Noisy, Test-Clean, and Test-Noisy. Dev-Clean and Dev-Noisy were evaluated to see how well each model was trained. As shown in the table, average CERs and WERs for all the ASR models were similar for both Dev-Clean and Dev-Noisy, except for ASR-only with clean-condition training. The CER and WER of ASR-only with clean-condition training were higher than those of other ASR models. This was because the Dev-Noisy dataset corresponded to a mismatched condition for ASR-only with clean-condition training. Specifically, the proposed optimization approach reduced the average CER and WER on the Dev-Noisy dataset by 0.26% and 0.27%, respectively, compared to the approach of separate optimization of speech enhancement and ASR. It was also revealed that the proposed optimization approach achieved a lower average CER and WER, by 0.30% and 0.46%, respectively, than the conventional optimization approach. Moreover, compared to the conventional two-step joint optimization approach, the proposed optimization approach provided average CER and WER reductions of 0.07% and 0.04%, respectively, for the Dev-Noisy dataset. 

Next, we compared the performance of ASR models evaluated on Test-Clean and Test-Noisy. Note that Test-Noisy was a matched noise condition for all the ASR models except for ASR-only with clean-condition training. Comparing the first and second rows of the table, ASR-only with multi-condition training achieved a lower average CER and WER than ASR-only with clean-condition training. In particular, the reduction in CER and WER was significant when Test-Noisy was evaluated. Next, it was shown that SE-ASR further reduced the average CER and WER on Test-Noisy by 0.06 % and 0.49%, compared to ASR-only with multi-condition training. However, the CER and WER of SE+ASR trained by the conventional joint optimization approach were slightly increased when the Test-Noisy dataset was evaluated. This implies that SE and ASR supported the distinct goals for speech quality and ASR performance, respectively, meaning that it could be difficult to guide the combined model towards the direction of ASR performance improvement. On the other hand, the conventional two-step joint optimization approach, as shown in the fifth row of the table, reduced the average CER and WER on the Test-Noisy dataset by 0.09% and 0.21%, respectively, compared to the joint optimization. As expected, SE+ASR trained by the proposed two-step joint optimization approach achieved the lowest CER of 9.07% and WER of 15.54% on the Test-Noisy dataset. Especially when compared to the conventional two-step joint optimization approach, the proposed optimization approach provided average CER and WER reductions of 0.05% and 0.23%, respectively. Since the main difference between the conventional and proposed optimization approaches was the ASR auxiliary loss in the first-step processing, it could be concluded, here, that the ASR auxiliary loss function could contribute to reducing CER and WER. 

### 4.3. Performance Evaluation under Mismatched Noise Conditions

Table 2 compares the average CER and WER of the ASR models trained by different optimization approaches under mismatched noise conditions. Compared to Table 1, average CERs and WERs for all the ASR models were increased without regard to any training approach. This was due to the mismatched noise conditions between the training and test datasets. Among the ASR models with speech enhancement, SE-ASR showed the largest increase in the CER and WER, compared to the CER and WER as shown in Table 1. This was because the pipeline was separately optimized with each objective function. Thus, the performance degradation of the front-end module under the mismatched condition might accelerate the aggravation in the performance of the back-end module [48]. In spite of the mismatched condition, SE+ASR trained by the proposed two-step joint optimization approach showed the lowest CERs of 11.57% and 9.87% for the Dev-Noisy-Mismatched and Test-Noisy-Mismatched datasets, respectively. In addition, it was shown that the proposed optimization approach achieved the lowest WERs of 19.68% and 17.49% for the Dev-Noisy-Mismatched and Test-Noisy-Mismatched datasets, respectively. In particular, the proposed two-step joint optimization approach reduced average CER and WER by 0.31% and 0.03% for the Dev-Noisy-Mismatched dataset, compared to the conventional two-step joint optimization. It was also shown that the proposed two-step joint optimization approach achieved lower average CER and WER by 0.13% and 0.24% for the Test-Noisy-Mismatched dataset than the conventional two-step joint optimization.

### 4.4. Ablation Study

The effectiveness of the proposed two-step joint optimization approach on each processing block in the conformer-transducer ASR model was examined through an ablation study. Table 3 compares the average CER and WER of the ASR models when various combinations of the processing blocks in the conformer-transducer-based ASR model were trained by the proposed two-step joint optimization approach. Note here that the CER and WER in the last row of this table are identical to those in the last row of Table 1. The ASR model in the first row of the table corresponds to the speech enhancement model trained only using the first-step processing of the proposed approach without applying the second-step processing. Compared to the ASR model with all the blocks trained using the proposed optimization approach, the ASR model without any training on the conformer-transducer had a higher CER and WER. Next, the proposed two-step joint optimization approach was applied to the encoder block only, the joint network only, and both blocks. It can be observed from the table that the ASR model with only the joint network trained by the proposed optimization approach achieved the lowest CER and WER on the Test-Noisy and Test-Noisy-Mismatched datasets. These results might have been obtained because the encoder block and joint network were located in the front part and the rear part of the conformer-transducer, respectively, as shown in Figure 2. In other words, as revealed in [49], the performance from the perspective of fine-tuning could be improved by freezing the front-part parameters of the neural network architecture and updating the weights of only the rear part. 

## 5. Conclusions

In this paper, a two-step joint optimization approach was proposed for training a pipeline composed of two different models: a speech enhancement model and an ASR model. In particular, a DCCRN-based speech enhancement model and a conformer-transducer-based ASR model were used as the front-end and the back-end of the pipeline, respectively. The first-step processing of the proposed joint optimization approach trained the front-end model only, whereas the second-step processing trained all the parameters of the combined model together. Therefore, the first-step processing needed to consider a new loss function to make the front-end model support the goal of the back-end model. To this end, an auxiliary ASR loss function was also proposed here for the first-step processing. 

Specifically, the procedure of the proposed two-step joint optimization approach started with the pre-training of the speech enhancement model as well as the ASR model, which were individually trained upon each task dataset. Then, in the first step, the enhanced speech was generated by applying the speech enhancement model to noisy speech, and the speech quality loss between the clean and enhanced speech was calculated. The clean and enhanced speech were separately fed to the encoder of the ASR model, and an auxiliary ASR loss was calculated. Finally, the parameters in the speech enhancement model were optimized using the speech quality and auxiliary ASR loss function. In the second step, all the model parameters in the speech enhancement and ASR models were updated together by using the speech quality and the ASR losses.

Three noteworthy results were observed in this study. First, conventional two-step optimization such as asynchronous subregion optimization demonstrated better ASR performance than traditional single-step joint optimization. Second, the proposed two-step joint optimization approach employing the auxiliary ASR loss function improved the ASR performance compared to the conventional two-step optimization that only used the speech quality loss function in the first-step. In addition, it was demonstrated by the ablation study that the proposed two-step joint optimization approach provided distinct effects on the processing blocks of the conformer-transducer ASR model.

In relation to future work, as described above, the proposed joint optimization approach achieved better performance when it was only applied to the joint network of the conformer-transducer ASR model. This implies that it is necessary to improve the encoder block of the conformer-transducer to generate a more feasible high-level representation feature by applying contrastive or metric learning [50,51,52]. Furthermore, the encoder block of the conformer-transducer could be integrated with the speech enhancement model because both should contribute towards speech representation for ASR, which will result in further improvement of the ASR performance. These ideas can be explored in future work.

## Figures and Tables

**Table 1:** Comparison of the average character error rate (CER) and word error rate (WER) of automatic speech recognition (ASR) models trained by various optimization approaches under clean and matched noise conditions.

Model	Training Approach	Dev-Clean		Dev-Noisy		Test-Clean		Test-Noisy	
		CER (%)	WER (%)	CER (%)	WER (%)	CER (%)	WER (%)	CER (%)	WER (%)
ASR-only	Clean-Condition Training	3.50	8.00	28.08	39.96	3.53	8.79	25.68	37.39
ASR-only	Multi-Condition Training	3.50	8.00	9.68	16.61	3.53	8.54	9.26	16.43
SE-ASR	Separate Optimization	3.50	7.91	9.57	16.04	3.51	8.42	9.20	15.94
SE+ASR	Joint Optimization	3.48	7.98	9.61	16.23	3.51	8.41	9.21	15.98
SE+ASR	Conventional Two-Step Joint Optimization	3.49	7.91	9.38	15.81	3.50	8.37	9.12	15.77
SE+ASR	Proposed Two-Step Joint Optimization	3.48	7.92	9.31	15.77	3.50	8.37	9.07	15.54

**Table 2:** Comparison of the average character error rate (CER) and word error rate (WER) of automatic speech recognition (ASR) models trained by various optimization approaches under mismatched noise conditions.

Model	Training Approach	Dev-Noisy-Mismatched		Test-Noisy-Mismatched	
		CER (%)	WER (%)	CER (%)	WER (%)
ASR-only	Clean-Condition Training	27.67	37.20	24.46	34.37
ASR-only	Multi-Condition Training	12.26	20.01	10.46	18.28
SE-ASR	Separate Optimization	16.42	28.67	16.39	28.44
SE+ASR	Joint Optimization	12.30	20.81	10.19	17.80
SE+ASR	Conventional Two-Step Joint Optimization	11.88	19.71	10.00	17.61
SE+ASR	Proposed Two-Step Joint Optimization	11.57	19.68	9.87	17.37

**Table 3:** Ablation study of the proposed two-step joint optimization approach applied to various combinations of the processing blocks in the conformer-transducer speech recognition model, measured by average character error rate (CER) and word error rate (WER), where check symbol √ means the proposed two-step joint training approach being applied.

Speech Enhancement	Speech Recognition Block Trained in Second-Step Processing			Test-Clean		Test-Noisy		Test-Noisy-Mismatched	
	Encoder	Prediction Network	Joint Network	CER (%)	WER (%)	CER (%)	WER (%)	CER (%)	WER (%)
√				3.51	8.34	9.09	16.27	9.92	17.73
√	√			3.53	8.60	10.85	18.14	10.27	17.92
√			√	3.50	8.29	8.90	15.46	9.42	16.57
√	√		√	3.53	8.61	10.79	17.99	10.18	17.90
√	√	√	√	3.50	8.37	9.07	15.54	9.87	17.49

## Data Availability

Publicly available datasets: LibriSpeech: https://www.openslr.org/12 (accessed on 5 June 2022). Deep Noise Suppression challenge: https://github.com/microsoft/DNS-Challenge/tree/interspeech2020/master (accessed on 5 June 2022).
